# Microstructural Analysis of Hot-Compressed Mg-Nd-Zr-Ca Alloy with Low Rare-Earth Content

**DOI:** 10.3390/ma18194490

**Published:** 2025-09-26

**Authors:** Yiquan Li, Bingchun Jiang, Rui Yang, Lei Jing, Liwei Lu

**Affiliations:** 1 School of Mechanical and Electrical Engineering, Guangdong University of Science and Technology, Dongguan 523083, China; liyiquan2025@163.com; 2 Sanya Institute of Hunan University of Science and Technology, Sanya 572024, China; 18259037438@163.com (R.Y.); cqullw@163.com (L.L.); 3 Northwest Nonferrous Metal Baoji Innovation Research Institute, Baoji 721000, China; zfjinglei@163.com

**Keywords:** Mg-Nd-Zr-Ca alloy, hot compression, continuous dynamic recrystallization, dynamic precipitation

## Abstract

Microstructural analysis of hot-compressed magnesium alloys is crucial for understanding the plastic formability of magnesium alloys during thermo-mechanical processing. Thermal compression tests and finite element simulations were conducted on a low rare-earth (RE) Mg-1.8Nd-0.4Zr-0.3Ca alloy. Multiple microstructural characterization techniques were employed to analyze slip systems, twinning mechanisms, dynamic recrystallization (DRX), and precipitate phases in the hot-compressed alloy. The results demonstrated that the equivalent strain distribution within compressed specimens exhibits heterogeneity, with a larger equivalent strain in the core. After thermal compression, the original microscopic structure formed a necklace-like structure. The primary DRX mechanisms comprise continuous dynamic recrystallization (CDRX), twin-induced dynamic recrystallization (TDRX), and particle-stimulated nucleation (PSN). Pyramidal slip and recrystallization constitute primary contributors to peak texture weakening and tilting. Mg_4__1_Nd_5_ and α-Zr phases enhanced dislocation density by impeding dislocation motion and promoting cross-slip activation. Hot compression provided the necessary thermal activation energy and stress conditions for solute atom diffusion and clustering, triggering dynamic precipitation of Mg_4__1_Nd_5_ phases.

## 1. Introduction

Magnesium alloys have demonstrated broad application prospects in lightweight transportation equipment due to their high specific strength and weight reduction advantages. In recent years, Mg-Nd, Mg-Gd, and Mg-Y series rare earth (RE) magnesium alloys have become a research focus owing to their significantly enhanced mechanical properties and thermal stability [1,2,3]. For instance, Zhang et al. [4] discovered that the addition of 3 wt.% Nd to the ZK60 alloy effectively enhances mechanical properties through the mechanisms of precipitation strengthening and texture strengthening. Wang et al. [5] reported concurrent improvements in yield strength, tensile strength, and elongation for Mg-Gd-Y-Zn-Zr alloys processed by sequential extrusion and controlled rolling. These enhancements principally result from substantial grain refinement and texture modification. Furthermore, Zhang et al. [6] demonstrated RE-enhanced strengthening in Mg-13Gd alloys via integrated thermo-mechanical processing, achieving a yield strength of 458 MPa and an ultimate tensile strength of 493 MPa, which originates from Gd-induced grain refinement and precipitation hardening. However, conventional RE magnesium alloys frequently rely on high RE contents to enhance strength and ductility, while the associated cost implications restrict their widespread application. Consequently, the development of low RE magnesium alloys has garnered significant attention.

In the development of low-RE magnesium alloys, non-rare earth elements such as Zr and Ca have been extensively investigated as cost-effective alternatives to partially replace RE elements, thereby reducing material costs while maintaining or even enhancing mechanical properties. Zr acts as a potent grain refiner, significantly enhancing strength by promoting nucleation and inhibiting grain growth during dynamic recrystallization [7]. Xu et al. [8] demonstrated that simultaneous addition of Zr and RE elements effectively promotes the precipitation of secondary phases in Mg-Zn-Zr alloys, achieving superior mechanical properties. Additionally, Kang et al. [9] found that adding Ca to Mg-2Zn alloys refines recrystallized grains, alters the size and distribution of alloy precipitates, and strengthens texture, leading to increased strength and reduced elongation. Wang et al. [10] discovered that adding a small amount of Ca to Mg-Zn-La alloys similarly refines grain size, promotes precipitation, and thereby enhances strength and ductility. These findings highlight the potential of Zr and Ca as economical alternatives to RE elements in magnesium alloy design.

The effect of thermal deformation on microstructure is a critical factor determining the formability and mechanical properties of magnesium alloys. To facilitate the development of novel high-performance magnesium alloys, researchers have extensively studied microstructural mechanisms including slip, twinning, dynamic recrystallization (DRX), and precipitation [11,12]. For example, Wang et al. [13] employed DEFORM-3D software to conduct finite element simulations of the hot compression of a Mg-Gd-Y-Zr alloy, with results showing strong agreement with experimental data. Kang et al. [14] found that pre-aging treatment influences the nucleation and growth of {10−12} twin structures in extruded Mg-6Zn-1Gd-1Er alloys, leading to the formation of stronger fiber textures by inhibiting DRX and thereby improving mechanical properties. Zhou et al. [15] used electron back-scattered diffraction (EBSD) to study high-strength and tough Mg-Er extruded alloys and found that the addition of Mn promotes the activation of multiple slip systems. Zhang et al. [16] further investigated the precipitation behavior and mechanical properties of a novel Mg-Nd-Sm-Zn-Zr alloy using transmission electron microscope (TEM) and first-principles calculations. Despite these advances, the microstructure and deformation mechanism of hot-compressed low-RE Mg-Nd-Zr-Ca alloys remain unclear. In particular, the effects of precipitates on dislocation motion, recrystallization nucleation, and grain growth require systematic investigation.

This study focuses on a low-RE Mg-1.8Nd-0.4Zr-0.3Ca alloy, employing hot compression tests and finite element simulations combined with multiscale characterization techniques, such as EBSD and TEM, to systematically investigate the synergistic mechanisms among slip, twinning, DRX, and precipitates. Special emphasis is placed on examining how various deformation mechanisms influence microstructural evolution and texture development. The work aims to elucidate slip system characteristics between recrystallized grains and deformed matrices during DRX processes, while highlighting the role of precipitation in dislocation activity and dynamic recrystallization. These findings are expected to provide theoretical foundations for optimizing the thermomechanical processing of low-RE magnesium alloys.

## 2. Experimental Procedure

**Figure 1:**
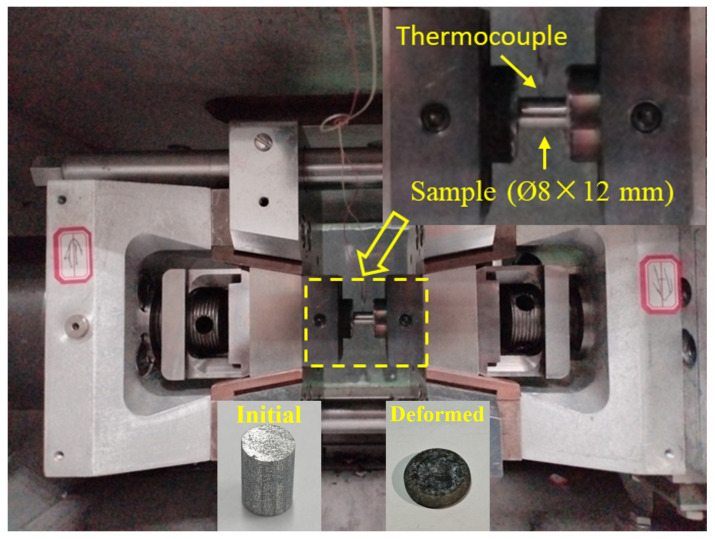
Schematic of the hot compression experimental setup.

This study employed an Mg-1.8Nd-0.4Zr-0.3Ca alloy, whose nominal chemical composition comprised 1.8 wt% Nd, 0.4 wt% Zr, and 0.3 wt% Ca, and balance Mg. The starting material of the Mg-1.8Nd-0.4Zr-0.3Ca alloy was in the form of as-cast ingots with a diameter of 70 mm, supplied by Hunan Rongtuo New Material Research Co., Ltd., Xiangtan, China. To eliminate the influence of initial casting surface defects, the outer layer of the ingot was removed by machining prior to processing, resulting in a final specimen with dimensions of 60 mm in both diameter and length. The as-cast specimens were subjected to solid solution treatment at 525 °C for 12 h, followed by water quenching to room temperature. Subsequently, the solution-treated samples were machined into cylindrical specimens measuring 8 mm in diameter and 12 mm in height via wire electrical discharge machining to ensure dimensional accuracy and minimize microstructural alterations. As shown in Figure 1, hot compression tests were conducted using a Gleeble-3500 thermo-mechanical simulator (Data Sciences International, Inc., St. Paul, MN, USA) with a deformation temperature of 430 °C and a strain rate of 0.03 s^−^^1^. Graphite sheets were placed between the specimen and anvils to minimize interfacial friction. The specimen temperature was regulated by a thermocouple welded to its surface. Samples were heated to the target temperature at 10 °C/s and held for 5 min, then compressed to a true strain of 0.6, followed by immediate water quenching to preserve the deformed microstructure.

**Figure 2:**
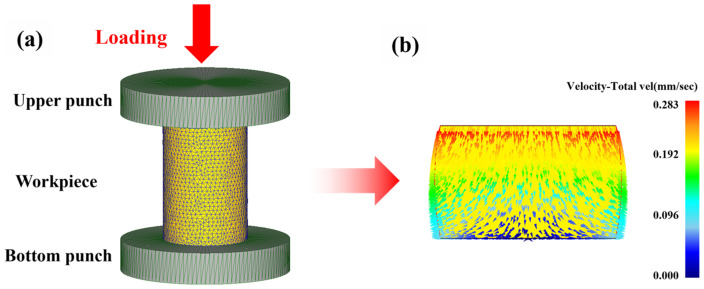
Finite element simulation of alloy hot compression: (**a**) Simulation model, (**b**) Flow velocity distribution on longitudinal section of deformed specimen at true strain 0.6.

As illustrated in Figure 2a, a geometric model identical to experimental dimensions was established using DEFORM-3D (Version 11.0) finite element simulation software, and a three-dimensional finite element simulation of the hot compression process was conducted. The primary parameters of the materials and processes used in the computer simulation are listed in Table 1. To establish the material model for the Mg-1.8Nd-0.4Zr-0.3Ca alloy, the true flow stress data dependent on strain, strain rate, and temperature were imported, adopting a von Mises yield function with a Poisson’s ratio of 0.35. The upper and lower punches were defined as rigid bodies, while the magnesium alloy specimen was modeled as a plastic body and discretized into 32,000 tetrahedral elements using adaptive meshing. During hot compression, the bottom punch remained stationary, whereas the upper punch moved downward along the negative Y-axis direction. The shear friction coefficient between the specimen and the punch was set to 0.3, with a contact tolerance of 0.0002 mm. The ambient temperature was set to 20 °C, while the compression temperature was maintained at 430 °C. An upper punch velocity of 0.27 mm/s was applied to correspond to hot compression at 0.03 s^−^^1^ strain rate, with simulation steps set to 60 and a step length of 0.09 mm. The deformation process was simulated using the Lagrangian incremental method, with the conjugate gradient method employed as the solver and the direct iterative method utilized for the iteration procedure. The velocity tolerance was set to 0.005, and the force tolerance was set to 0.05. The flow distribution on the longitudinal section of the deformed specimen at 0.6 true strain obtained from simulation is presented in Figure 2b.

The as-solution specimen and deformed compression specimen were sectioned in a manner parallel to the compression direction (CD). The central region of the cross-section was designated as the microstructure observation area. During the metallographic preparation process, all samples were sequentially mechanically ground using silicon carbide abrasive papers with progressively finer grits of #300, #800, #1200, and #2000. They were then rough polished with a 1 μm diamond suspension, followed by final precision polishing using a 0.05 μm colloidal silica suspension to obtain a scratch-free surface. The as-solution specimens were etched for 5–15 s using a solution composed of 4.2 g picric acid, 10 mL acetic acid, 70 mL ethanol, and 10 mL distilled water. The microstructure of the etched as-solution specimens was subsequently characterized using an inverted optical microscope (OM) manufactured by Olympus Corporation (Hachioji-shi, Tokyo, Japan) and a Tescan Mira4 field emission scanning electron microscope (SEM). The as-solution and hot-compressed samples were mechanically ground, polished, and examined by X-ray diffraction (XRD) using a SmartLab-9kW X-ray diffractometer (Rigaku Corporation, Osaka, Japan) with Cu Kα radiation. The scans were performed from 20° to 80° at a rate of 5° per minute to identify the phase characteristics of the samples. The hot-compressed samples after mechanical grinding and polishing were electro-polished by the AC2 solution. Then the microstructure was characterized using an EBSD detector (Oxford Symmetry, Oxford, UK) with a scanning electron microscope operating at 15 kV, a step size of 0.8 μm, and a sample tilt of 70°. Key microstructural parameters, including misorientation, inverse pole figure (IPF) maps, and texture components, were extracted from EBSD datasets using HKL Channel 5 (Version 5.0). For TEM characterization, the hot-compressed samples were first mechanically ground into 200 μm thick slices, and 3 mm diameter discs were punched from these slices. The discs were then subjected to twin-jet electropolishing in a 4.5% perchloric acid ethanol solution at 35 V and −45 °C, followed by additional thinning using a precision ion polishing system at −100 °C. Microstructural characterization was subsequently conducted using a JEM-F200 Multi-purpose Electron Microscope (ThermoFisher, Hilsboro, CA, USA) operated at 200 kV.

## 3. Results

### 3.1. Finite Element Simulation

**Figure 3:**
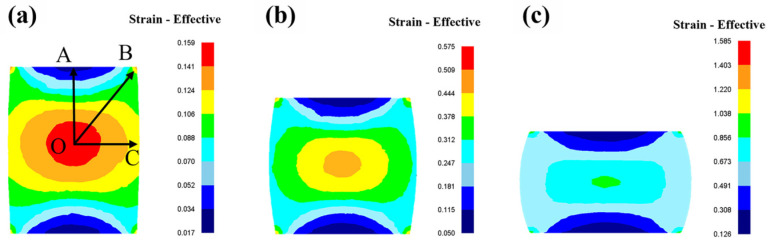
Distribution of equivalent plastic strain in Mg-Nd-Zr-Ca alloy during hot compression at true strains of: (**a**) 0.1, (**b**) 0.3, (**c**) 0.6.

Figure 3 displays the effective strain distribution within the Mg-Nd-Zr-Ca alloy subjected to different deformation levels. Effective strain values exhibit significant regional variations, concentrating at the specimen core (point O). Distinct local loading patterns explain this phenomenon [13]. The core region microstructure undergoes lateral expansion and material flow under triaxial compressive stresses, while interfacial friction between the specimen and the anvil induces shear deformation at the base. Prior investigations confirm that increased equivalent plastic strain in metallic materials under hot deformation conditions corresponds to greater stored energy [17]. Accumulating adequate deformation energy is essential for initiating DRX. Elevated equivalent plastic strain zones deliver more energy, playing a vital role in activating DRX and refining microstructures.

Furthermore, the uniformity of effective strain distribution directly reflects grain size homogeneity. To quantify strain distribution uniformity in magnesium alloy specimens post hot compression, values of *ε_min_*, *ε_max_*, and *ε_avg_* were extracted from cross-sections using DEFORM-3D software. The inhomogeneity index *C_i_* [18] was calculated via Equation (1):

(1) $$C_{i}=\frac{\varepsilon_{max}-\varepsilon_{min}}{\varepsilon_{avg}}$$

where *ε_min_*, *ε_max_*, *ε_avg_* denote minimum, maximum, and average effective strains, respectively. Summarized *ε_min_*, *ε_max_*, *ε_avg_*, and *C_i_* values are tabulated in Table 2. As listed, *C_i_* increases from 1.361 at true strain 0.1 to 1.595 at true strain 0.6, demonstrating enhanced strain inhomogeneity with progressive deformation.

### 3.2. Microstructure

**Figure 4:**
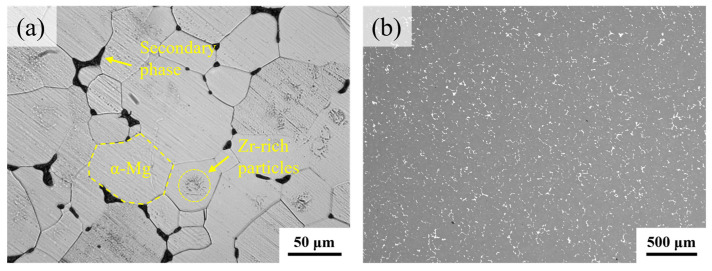
Microstructure of Mg-Nd-Zr-Ca alloy in solid solution: (**a**) OM, (**b**) SEM.

Figure 4 presents the microstructural characteristics of Mg-Nd-Zr-Ca alloy in solution-treated. It has been observed that following solid solution treatment, the major portion of the precipitated phase is dissolved within the equiaxed α-magnesium matrix, with a negligible amount of network-like secondary phase distributed at the grain boundaries. Notably, fine particles with annular distributions are observed within grains, a common feature in Zr-containing rare-earth Mg alloys, where these aggregated precipitates are typically Zr-rich particles [19].

**Figure 5:**
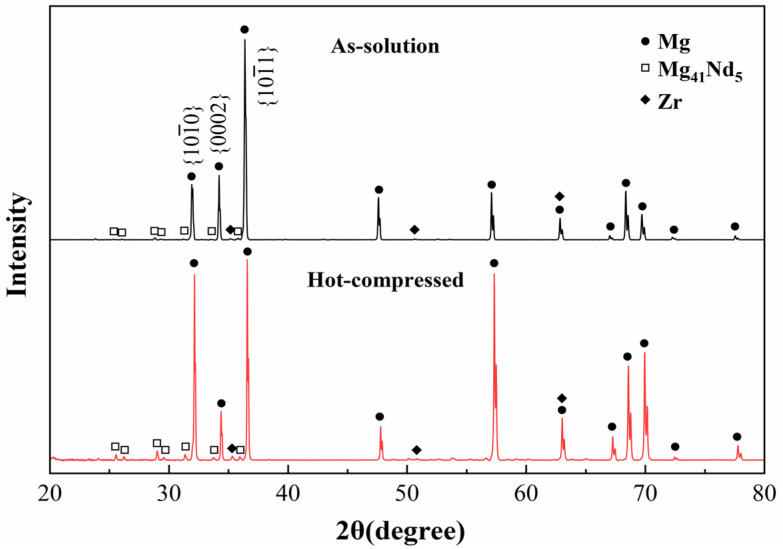
XRD patterns of solution-treated and hot-compressed Mg-Nd-Zr-Ca alloy specimens.

Figure 5 presents XRD patterns of the Mg-Nd-Zr-Ca alloy after solution treatment and hot compression. Peak identification confirms secondary phases of Mg_4__1_Nd_5_ and α-Zr in the solution-treated sample. After hot compression, the peak position remained unmodified, while the diffraction intensity of Mg_41_Nd_5_ increased. These results indicate that no phase transformation occurred during the hot compression process and that the Mg_41_Nd_5_ phase was dynamically precipitated. Notably, the {10−10} diffraction peak of the Mg matrix intensifies significantly after compression, exceeding the {0002} basal peak intensity compared to the solution-treated sample. This suggests alterations in grain orientation during compression [20].

**Figure 6:**
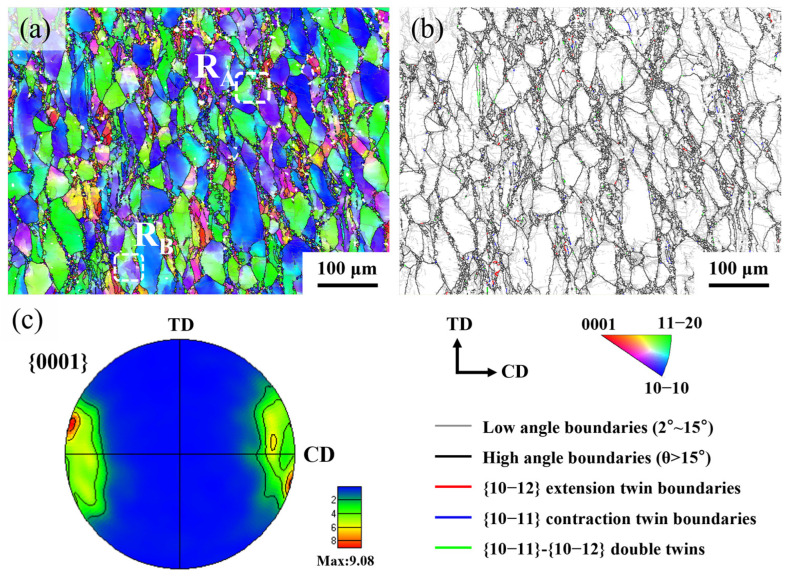
EBSD analysis of hot-compressed Mg-Nd-Zr-Ca specimen: (**a**) IPF maps, (**b**) Grain boundary map, (**c**) {0001} pole figures.

To investigate the microstructure of Mg-Nd-Zr-Ca alloy after hot compression, systematic characterization was performed using EBSD. The IPF maps, grain boundary distributions, and {0001} pole figures of hot-compressed specimens are shown in Figure 6a–c, respectively. Observation reveals that the original equiaxed coarse grains were significantly elongated perpendicular to the CD, with fine equiaxed DRX grains distributed near original grain boundaries forming necklace structures. Pronounced crystallographic orientation gradients and abundant low-angle grain boundaries (LAGB) were detected within coarse grains. Lv et al. [21] reported analogous features in hot-compressed ZM60 alloy, attributing their formation to CDRX mechanisms. In addition to conventional grain boundaries, a small number of lenticulars {10−12} extension twin boundaries (ET), {10−11} contraction twin boundaries (CT), and {10−11}-{10−12} double twins (DT) were observed, as shown in Figure 6b, indicating significant activation of twinning mechanisms during deformation. Owing to the limited availability of slip systems in the crystal structure of Mg alloys, twinning served as a crucial mechanism for accommodating plastic deformation. When the applied stress exceeded the critical resolved shear stress for twinning, {10−12} tensile twins preferentially nucleated in grains with c-axis tension. With progressively increasing strain, {10−11} contraction twins and {10−11}-{10−12} double twins were subsequently activated to accommodate larger plastic strains [22,23]. Figure 6c displays the {0001} pole figure of the hot-compressed alloy. The pole density maxima of basal planes concentrate near the CD axis with peak intensities of 9.08, demonstrating a distinct CD//⟨0001⟩ texture. Notably, angular deviations exist between pole density maxima and the CD axis. During hot compression, basal slip in original grains readily develops basal texture perpendicular to deformation direction, whereas non-basal slip, twinning, and DRX modify texture intensity and variant types [24].

## 4. Discussion

### 4.1. Dynamic Recrystallization Mechanism

**Figure 7:**
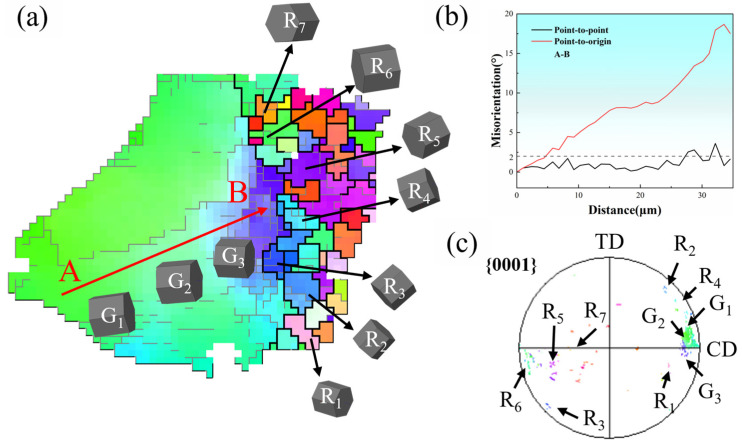
DRX characteristics of region R_A_ in Figure 6a: (**a**) Inverse pole figure map, (**b**) Line profiles of the misorientation angle along red arrow AB, (**c**) {0001} pole figure.

In order to investigate the dynamic recrystallisation mechanism of the hot-compressed Mg-Nd-Zr-Ca alloy, the typical DRXed region R_A_ and R_B_ were selected from Figure 6a for further analysis. As shown in Figure 7, region R_A_ in Figure 6a was magnified to investigate DRX evolution in Mg-Nd-Zr-Ca alloy under isothermal compression. As shown in Figure 7a, abundant LAGBs are present in the parent grains, with the density of LAGBs gradually increasing from point A to point B, and many DRX grains forming near point B. The inserted three-dimensional diagram indicates that the orientation within the parent grain undergoes continuous changes. Figure 7b shows the line profile along the red arrow AB in Figure 7a, with the orientation difference angle from point to origin gradually increasing to approximately 17°, indicating significant deformation and dislocation pile-up within the parent grain. Additionally, the {0001} pole figure in Figure 7c shows that the c-axis of the dynamic recrystallization is tilted relative to the parent crystal. This is the typical formation mechanism of CDRX. The formation mechanism of CDRX can be summarized into the following key steps: First, dislocations proliferate continuously through slip and climb mechanisms during thermal deformation, forming regions of high dislocation density. Subsequently, dislocation rearrangement and annihilation form subgrain structures, establishing a LAGB network within the grains or near the original grain boundaries. As strain continues to increase, subgrains gradually accumulate orientation differences through lattice rotation, ultimately transforming into high-angle grain boundaries (HAGB) and forming fine equiaxed DRX grains [25].

**Figure 8:**
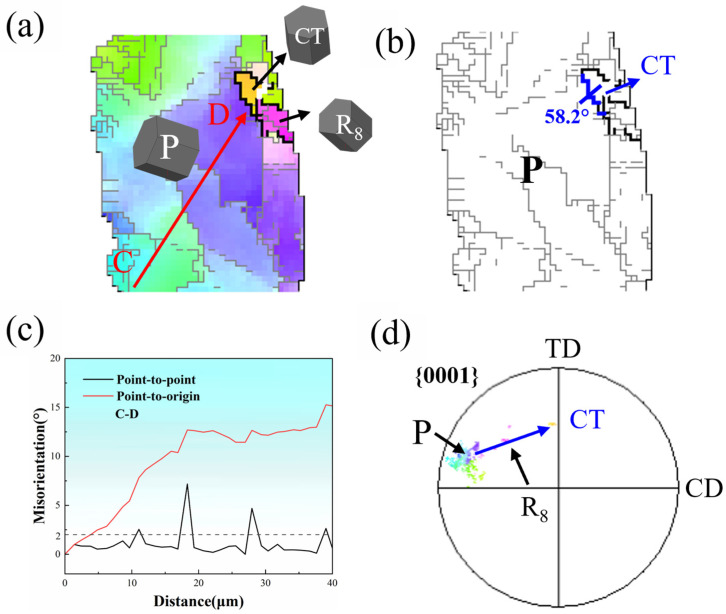
DRX characteristics of region R_B_ in Figure 6a: (**a**) Inverse pole figure map, (**b**) Grain boundary map, (**c**) Line profiles of the misorientation angle along red arrow CD, (**d**) {0001} pole figure.

Figure 8 analyzes region R_B_ to investigate twin effects on DRX behavior in magnesium alloys. Figure 8a reveals a {10−11} compression twin (CT) and DRX grain R_8_ within parent grain P. A discernible color gradient change manifests along the arrow CD in parent grain P. Figure 8c demonstrates that point-to-origin misorientation angles progressively exceed 15° along line CD, indicating substantial dislocation activity altering local orientations. Abundant LAGBs appear near twin boundaries, signifying severe dislocation pile-ups at twin junctions. When local strain surpasses a critical threshold, R_8_ grains assimilate surrounding dislocations to nucleate and grow, a process termed the twin-induced dynamic recrystallization (TDRX). However, intensive dislocation generation simultaneously increases stored energy and disrupts twin boundary integrity, causing twin density reduction [26]. Texture analysis further confirms significant orientation deviations between twins, twin-induced recrystallized grains, and parent grain P, as shown in Figure 8d. Collectively, twins effectively impede dislocation motion, elevate dislocation density, and promote DRX, thereby weakening basal texture intensity.

**Figure 9:**
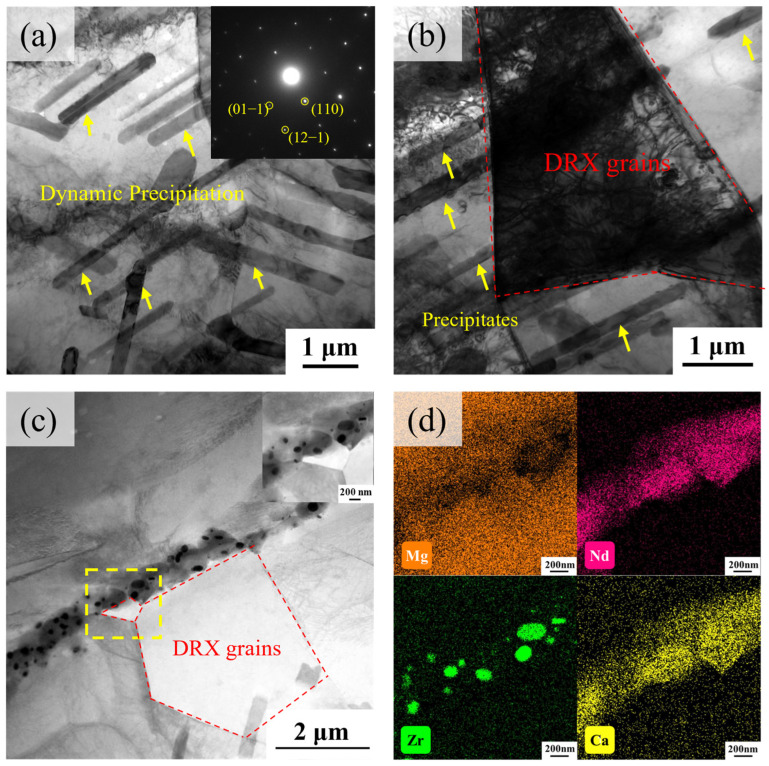
TEM micrographs of deformed specimens: (**a**–**c**) Distribution of precipitates and DRX grains; (**d**) the elemental maps corresponding to the boxed region in (**c**), showing Mg, Nd, Zr, and Ca.

TEM investigations can clarify the pivotal function of secondary-phase particles in governing dislocation behavior and DRX processes. Figure 9 presents bright-field TEM images and corresponding energy dispersive spectrometer (EDS) elemental mapping results of the hot-compressed samples. High-density rod-shaped secondary phases observed in Figure 9a were identified as Mg_4__1_Nd_5_ through selected-area electron diffraction characterization, with approximate lengths of 3 μm. These rod-shaped precipitates were not observed in previous microstructural analyses of solution-treated samples, indicating their formation through strain-induced precipitation mechanisms during hot compression. Plastic deformation during hot compression significantly increases dislocation density, which accelerates solute atom diffusion and facilitates localized enrichment and precipitation of solutes [27]. Additionally, granular precipitates (diameter < 1 μm) distributed along grain boundaries are observed in Figure 9c. As shown in Figure 9d, TEM-EDS analysis of these grain boundary precipitates confirms the solute segregation phenomenon of Nd, Ca, and Zr along grain boundaries.

Figure 9b,c reveal equiaxed recrystallized grains adjacent to precipitates. Pang et al. [28] proposed that precipitates act as nucleation sites for recrystallization, while the formation of recrystallized grains promotes dynamic precipitation. It is inferred that dispersed second-phase particles in Mg-Nd-Zr-Ca alloys impede dislocation motion during deformation, leading to dislocation accumulation around these particles and localized high strain energy. When accumulated energy reaches a critical threshold, DRX is triggered at second-phase interfaces via the particle-stimulated nucleation (PSN) mechanism. Newly formed grains facilitate solute atoms (e.g., Nd, Ca) diffusing from the supersaturated matrix and segregating, thereby promoting dynamic precipitation. However, these dynamic precipitates impede further growth of DRX grains, resulting in a low fraction of DRX grains observed in the EBSD analysis.

### 4.2. Texture Evolution

**Figure 10:**
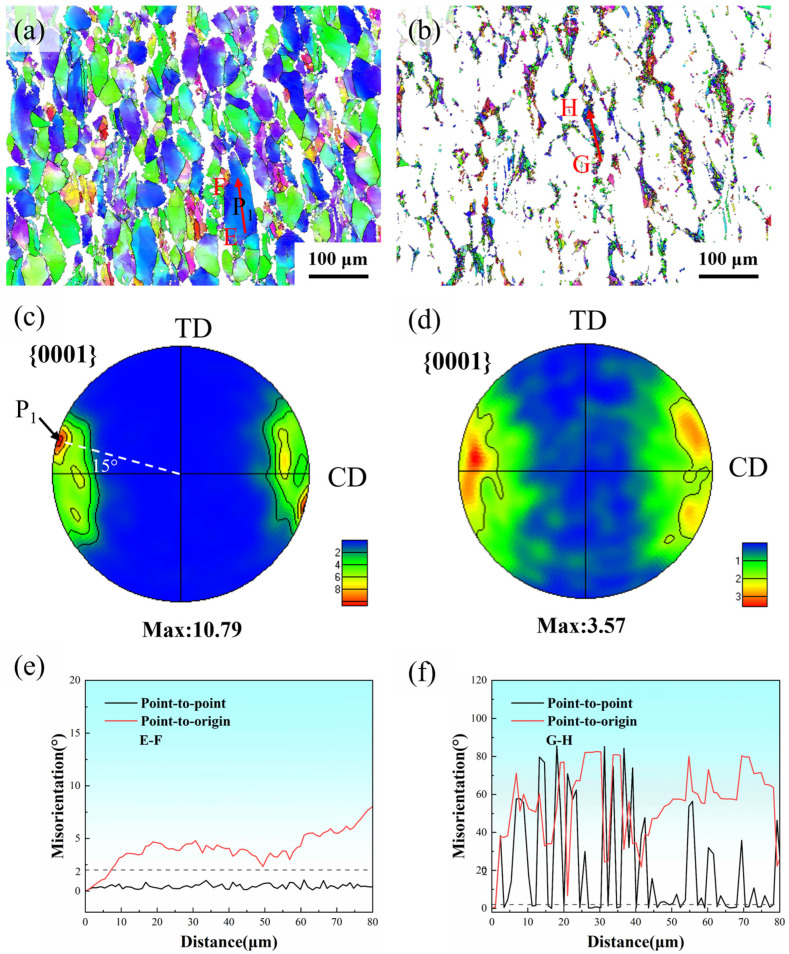
Inverse pole figure maps, {0001} pole figures, and misorientation angle for distinct grain types: Deformed grains (**a**,**c**,**e**); DRX grains (**b**,**d**,**f**).

To investigate the effects of fine DRX grains and coarse deformed grains on deformation mechanisms in Mg-Nd-Zr-Ca magnesium alloy, systematic analyses of IPF maps, pole figures, and misorientation angle distributions were conducted for both DRX grains and deformed grains, as shown in Figure 10. Figure 10c demonstrates that projections of coarse deformed grains on the {0001} pole figure concentrated at both ends of the CD, achieving a maximum pole density (MPD) of 10.79 and exhibiting typical <0001>//CD texture characteristics. It is noteworthy that grain P_1_ is situated at the point of MPD, with its c-axis deviating by 15° from the CD axis. As shown in Figure 10e, the accumulated point-to-origin misorientation angle along the red arrow EF in grain P_1_ gradually increases to 8.1°, indicating pronounced slip deformation within the coarse deformed grain. However, the slight fluctuations in orientation gradient suggest limited lattice distortion energy stored in these coarse grains, which is unfavorable for DRX formation. It is well established that basal slip in magnesium alloys possesses relatively low critical resolved shear stress, making it preferentially activated during hot deformation. Basal slip induces rotation of grain c-axes toward the CD, ultimately forming a strong <0001>//CD basal texture [29]. Figure 10d shows a highly dispersed distribution of fine DRX grains in the {0001} pole figure, with the MPD decreasing to 3.57. Figure 10f exhibits significant fluctuations in point-to-point misorientation angles along arrow GH, confirming substantial orientation differences between adjacent DRX grains, which further verifies the effectiveness of DRX grains in weakening basal texture.

### 4.3. Slip Systems and Dislocation Mechanisms

**Figure 11:**
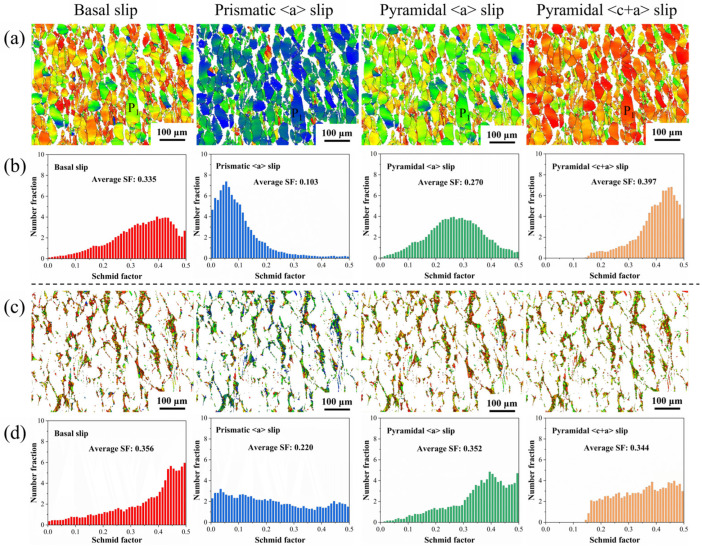
Schmid factor distributions and histograms for different slip systems: Deformed grains (**a**,**b**); DRX grains (**c**,**d**).

Figure 11 presents Schmid factor (SF) maps and corresponding distribution histograms of common slip systems in magnesium alloys for both DRXed grains and coarse deformed grains. Slip systems with SF values approaching 0.5 are considered easily activated, while grain orientations with SF > 0.3 are classified as soft orientations. As observed in Figure 11b, the mean SF values for basal slip and pyramidal <c+a> slip in deformed grains are 0.335 and 0.397, respectively, exceeding 0.3, while prismatic slip exhibits a significantly lower mean SF of 0.103. This indicates predominant activation of basal and pyramidal <c+a> slip systems within deformed grains. Extensive research demonstrates that rare earth elements in solid-solution form significantly enhance the activity of pyramidal <c+a> slip. The activation of the pyramid <c+a> slip causes the texture peak to shift from CD to TD, forming a double-peak texture [30]. Consequently, pyramidal slip activation is the primary reason for the 15° deviation of the c-axis from the CD axis in grain P_1_. Figure 11c shows the basal slip of recrystallized grains, with both the pyramidal <a> slip and the pyramidal <c+a> slip exhibiting high Schmid factors, indicating active dislocation activity. During thermal deformation, non-basal slip provides additional plastic deformation mechanisms for grains, coordinating local strain distribution and reducing stress concentration, thereby delaying crack initiation. Additionally, the local lattice rotation within grains caused by these slips promotes CDRX, leading to deviation of the c-axis from the CD and weakening the basal texture.

**Figure 12:**
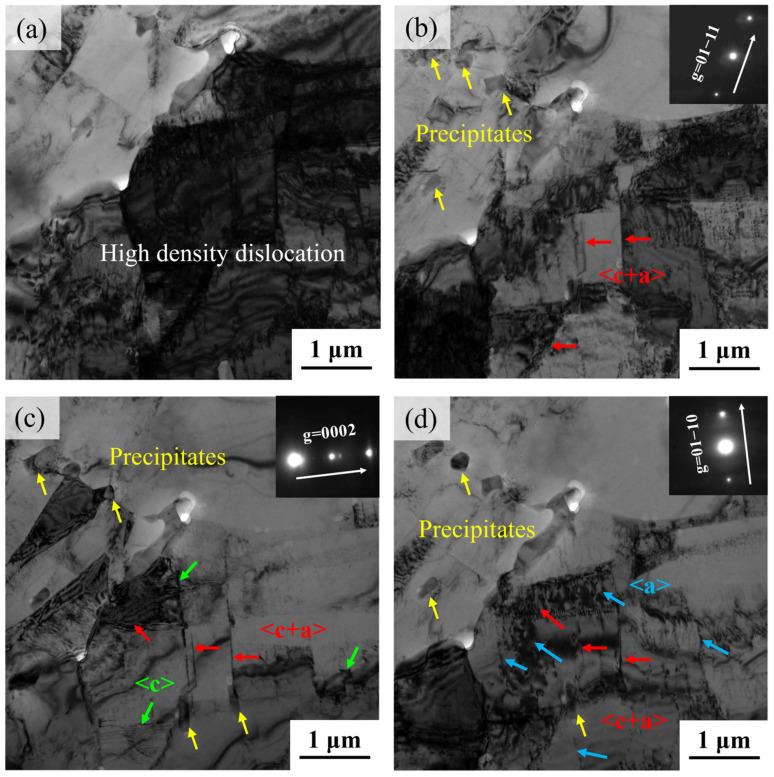
Two-beam bright-field TEM micrographs of deformed sample: (**a**) high density dislocation, (**b**) g = 01−11, (**c**) g = 0002, (**d**) g = 01−10.

As shown in Figure 12a, high-density dislocations were observed in the TEM microstructure of the hot-compressed sample. To identify active slip systems, the incident beam direction was set to B = [2−1−10], and TEM micrographs under different diffraction vectors (g) were acquired using two-beam diffraction conditions, as shown in Figure 12b–d. The invisibility criterion g·b=0 (where b denotes the Burgers vector) was employed to determine slip system types. When g = 01−11, all <a>, <c>, and <c+a> type dislocations remained visible. Under g = 01−10, <a> type dislocations became invisible due to satisfaction of the extinction criterion, while <c> type dislocations disappeared at g = 0−110. Through comparative analysis of TEM micrographs under varying g vectors, dislocation types were conclusively identified. Dislocation characterization revealed activated <c> and <c+a> type dislocations during hot deformation, consistent with prior EBSD analysis.

Notably, rod-shaped precipitates were observed near dislocation lines. Acting as obstacles to dislocation motion, these precipitates impede dislocation glide through a pinning effect, leading to dislocation accumulation around precipitates and consequently increasing dislocation density. Furthermore, these precipitates force dislocations to bypass second-phase particles via cross-slip, promoting the activation of non-basal slip in <c+a> dislocations, thereby significantly enhancing the deformation activation energy [31].

## 5. Conclusions

The microstructure of the hot-compressed Mg-1.8Nd-0.4Zr-0.3Ca alloy was systematically investigated through a series of hot compression tests, finite element simulations, and microstructural characterization techniques. The main findings are summarized as follows:

1. The distribution of equivalent strain in the hot-compressed specimens exhibited heterogeneity, with strain inhomogeneity increasing progressively with further deformation (*Ci* = 1.361–1.595). The central region of the specimen exhibited a high equivalent strain due to triaxial compressive stress, accumulating sufficient deformation energy to initiate DRX, which resulted in the formation of a necklace-like microstructure.
2. The predominant deformation mechanisms include basal slip, pyramidal slip, twinning, CDRX, TDRX, and PSN, which play critical roles in grain refinement and texture evolution. The deformed grains developed a strong <0001>//CD texture (MPD: 10.79) in the hot-compressed sample, whereas DRX grains effectively weakened the texture, reducing the MPD to 3.57. Basal and pyramidal <c+a> slips were predominantly activated in deformed grains, with average SF of 0.335 and 0.397, respectively. In contrast, DRX grains additionally activated pyramidal <a> slip, exhibiting an average SF of 0.352.
3. The high dislocation density in the hot-compressed sample, attributed to the pinning effect of precipitates on dislocations, induces the dynamic precipitation of the Mg_4__1_Nd_5_ phase. Dynamic precipitation promotes nucleation of DRX and inhibits grain growth of DRX grains, resulting in DRX grains with fine size.

## Figures and Tables

**Table 1:** Basic simulation parameters for hot-compressed.

Simulation and Material Parameters	Parameter Values
Workpiece length(mm)	12
Workpiece diameter(mm)	8
Poison’s ratio	0.35
Mesh type	Tetrahedral mesh
Total number of elements	32,000
Relative interference depth	0.7
Friction coefficient	0.3
Compression temperature (°C)	430
Upper punch velocity(mm/s)	0.27
Step length(mm)	0.09
Total simulation steps	60

**Table 2:** Values of ε_min_, ε_max_, ε_avg,_ and *C_i_*.

True Strain	ε_min_	ε_max_	ε_avg_	$C_{i}$
0.1	0.018	0.150	0.097	1.361
0.3	0.055	0.498	0.289	1.533
0.6	0.141	1.050	0.57	1.595

## Data Availability

The original contributions presented in this study are included in the article. Further inquiries can be directed to the corresponding author.

## Abbreviations

The following abbreviations are used in this manuscript:

RE	Rare earth
DRX	Dynamic recrystallization
PSN	Particle-stimulated nucleation
TDRX	Twin-induced dynamic recrystallization
EBSD	Electron back-scattered diffraction
TEM	Transmission electron microscope
OM	Optical microscope
SEM	Scanning electron microscope
CD	Compression direction
XRD	X-ray diffractometer
IPF	Inverse pole figure
LAGB	Low-angle grain boundary
ET	Extension twin
CT	Contraction twin
DT	Double twins
HAGB	High-angle grain boundary
EDS	Energy dispersive spectrometer
SF	Schmid factor
MPD	maximum pole density
